# THE ROLE OF PPARδ-DRIVEN β-OXIDATION IN BONE HEALTH DURING AGING

**DOI:** 10.1093/geroni/igac059.1611

**Published:** 2022-12-20

**Authors:** Matt Prideaux, Tom O'Connell, Yukiko Kitase

**Affiliations:** Indiana University, Indianapolis, Indiana, United States; Indiana University, Indianapolis, Indiana, United States; Indiana University School of Medicine, Indianapolis, Indiana, United States

## Abstract

Musculoskeletal disorders are a significant complication of aging, leading to increased morbidity and mortality. However, current understanding of the mechanisms by which aging affects skeletal health is limited. Osteocytes are the most numerous and long-lived bone cells and play key roles in maintaining bone mass by responding to anabolic signals such as mechanical loading. Energy metabolism is dysregulated in many cells with aging, however regulation of energy metabolism in osteocytes and how this is affected during aging and by mechanical loading remains undefined. To investigate this, we first used IDG-SW3 osteocyte cells to determine the effects of mechanical loading on osteocytes in vitro by applying fluid flow shear stress (FFSS). FFSS increased Pparδ and Cpt1 expression, key promoters of fatty acid β-oxidation (FAO). Pharmacological antagonism of PPARδ or CPT1 resulted in dysregulated expression of key bone remodeling genes and impaired ATP release in response to FFSS. In vivo, mechanical loading significantly increased FAO in tibia cortical bone. However, FAO was impaired in the bones from aged mice. To further elucidate the role of osteocyte FAO, we deleted PPARδ specifically in osteocytes (PPARδ cKO), which resulted in decreased FAO and bone volume in female PPARδ cKO mice. Lastly, treatment of aging mice with the PPARδ activator GW0742 resulted in significantly increased bone mineral content, density and trabecular bone volume. These findings suggest important functions of osteocyte energy metabolism during aging and with mechanical loading on bone and identify PPARδ-driven FAO as a novel therapeutic target for improving skeletal health with aging.

